# Visual abstracts: an innovative way to disseminate scientific Information

**DOI:** 10.1590/2175-8239-JBN-2019-0213

**Published:** 2020-02-21

**Authors:** José A. Moura-Neto, Miguel Carlos Riella

**Affiliations:** 1 Brazilian Journal of Nephrology, São Paulo, SP, Brasil

**Keywords:** Visual Abstract, Graphic Summaries, Social Media, Periodical, Scientific Journals, Resumos Gráficos, Mídias Sociais, Publicação Periódica, Revista Científica

## Abstract

Technological innovations often occur and make an impact on many industries. In academia, Visual Abstracts have been a trend and represent a creative and dynamic way to disseminate scientific knowledge. Although still rare in Brazil, more than 15 journals already use Visual Abstracts worldwide. This brief paper intends to present the concept and discuss the potential effectiveness of this innovative tool.

## RESUMO

Inovações tecnológicas ocorrem frequentemente e causam impacto em diversos setores. No meio acadêmico, *Visual Abstracts* têm sido uma tendência e representam uma forma criativa e dinâmica de divulgar o conhecimento científico. Apesar de ainda raros no Brasil, mais de 15 periódicos já utilizam Visual Abstracts no mundo. Este breve artigo pretende apresentar o conceito e discutir a potencial efetividade dessa ferramenta inovadora.

In a constantly changing world, many fields deal with disruptive innovations and sectoral rearrangements. The academic world is no exception. In 1994, a simple and elegant model was proposed to describe the five basic functions of scientific journals. They are, in order of importance, according to the author herself: building a collective knowledge base, disseminating information, validating research quality, distributing rewards, and building scientific communities.^1^ Almost three decades later, this model still remains current; However, technological features that interfere with these functions, such as the internet, smartphones, applications and social media, have been developed. The impact of these advances on academia is varied and generally positive. Social media, for example, perform two of the functions described in the 1990s model: disseminating information and building scientific communities^2^.

Although traditional and sometimes stagnant, the academic publishing industry is undergoing profound changes. To reduce production and distribution costs, some scientific journals have opted in recent years to publish papers online only. The open access mode, which allows free access to the manuscript without the need to purchase, is also increasingly common. There are around 10,000 open access journals available.^3^ In addition, the most impactful scientific journals already have smartphone apps, podcasts, and social media where they interact and share their published papers. In this context, a recent tool has been popularized: the Visual Abstract (VA) or Graphical Abstract.

VA is indeed a trend among world scientific journals. More than 15 journals currently use this tool, including the Journal of the American Society of Nephrology (JASN), Clinical Journal of the American Society of Nephrology (CJASN), Annals of Surgery, New England Journal of Medicine, Journal of Vascular Surgery and Kidney International. Most medical journals have adopted VA only in the last four years; Annals of Surgery was a pioneer, having started VA publication in July 2016, while CJASN began shortly thereafter in 2017. Although only recently disseminated, VAs have been used in one-off initiatives for just over a decade; the open access journal of chemistry Molecules, for example, has been using this tool since 2008.^4^^,^ 5

In Brazil, the journals that incorporated this tool are still rare. The Brazilian Journal of Nephrology (BJN) was a pioneer in this regard and began using VAs in its quarterly volumes since the first issue of 2019. In 2018, it published some VAs on time on the Scielo in Perspective blog and its social media.^6^^,^ 7 The BJN, founded 40 years ago in 1979, is the official scientific publication of the Brazilian Society of Nephrology. It is indexed to Scielo, Lilacs and Pubmed/Medline since 2010.

While some journals encourage the authors themselves to submit a VA associated with the accepted paper, others have exclusive editors responsible for the creation of the VA, such as CJASN and BJN.^8^ The academics in this group, usually younger than the Associated Editors, are called Social Media and Visual Abstract Editors.

The main goal of VA is to summarize the core idea or key results from the study in a single graph with concise information. Thus, the journal aims to draw attention to the paper and disseminate knowledge to readers in a more accessible way and in a shorter period. Perhaps, more important than understanding VA is define what this tool is not. It is definitely not intended to be a substitute for the traditional written summary or for reading the paper itself. The reader should avoid reaching conclusions through the VA alone, as it is intended only to assist in deciding whether to read the full paper. A single slide could not accurately represent the complexity of the paper, as it is impossible to include all the important information in it.^2^

**Figure 1:**
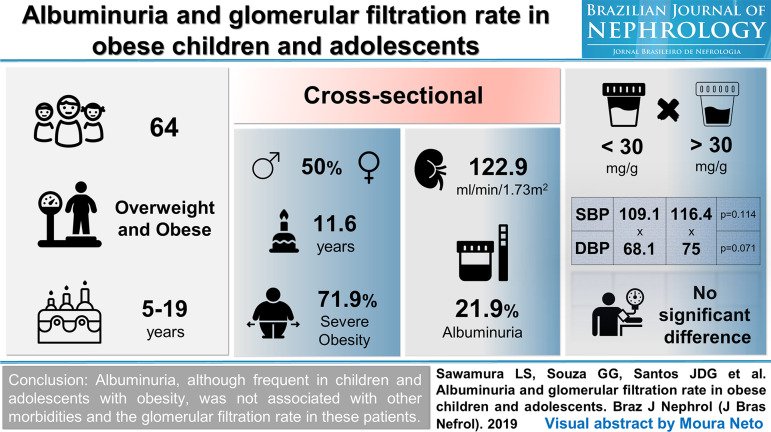
Visual Abstract published in the Brazilian Journal of Nephrology in 2019; 41(2):193-9.

**Figure 2:**
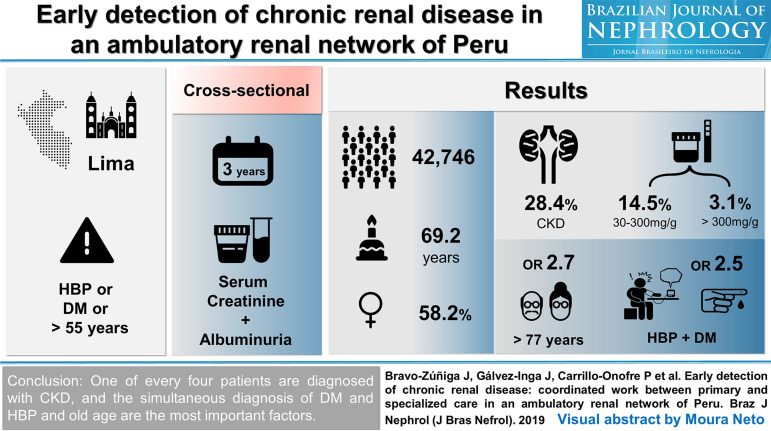
Visual Abstract published in the Brazilian Journal of Nephrology in 2019; 41(2):176-84.

**Figure 3:**
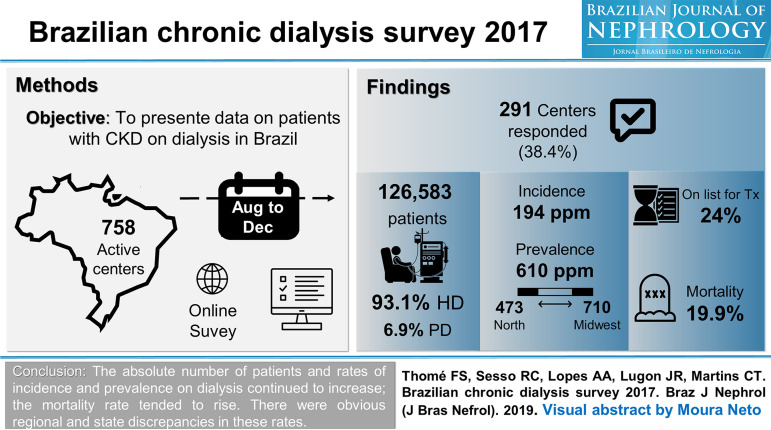
Visual Abstract published in the Brazilian Journal of Nephrology in 2019; 41(2):208-14.

The process of making the VA is variable and depends on the profile and skill (inspiration) of each VA Editor. There are manuals online to help prepare the VA in its various stages, with examples and the use of audiovisual aids.^9^^,^ 10 Even following the walkthrough, creativity is the key element in the process, and deciding what information will be available, and in no way it can be a trivial task. Attractive design counts almost as much as content. Finding the right balance of information, avoiding shortages and excesses, is perhaps the biggest challenge in making a VA. Figures 1, 2 and 3 are examples of VAs published by the BJN in 2019.

Each journal has specific VA-related guidelines, which define the format and layout, with the logo, as well as where and how mandatory elements such as the paper reference, and title should be included. Therefore, we see distinct styles between journals; "colorful" VAs from the New England Journal of Medicine’s have a very different style and pattern than those published in the CJASN, which bears more detailed information, or than those more concise VAs from Annals of Surgery.

In addition to content and design, choosing the media is also important, and there are a few options. It may be available on the magazine’s website, along with the paper’s link, or on the journal’s social media such as Twitter and Facebook. The power of social media is significant, even among academics. Many journals already have Social Media Editors in their editorial staff, although the roles and responsibilities of this group are not well defined.^11^ According to a recent study, when an paper is also posted on Twitter, it may have up to three times as many views.^12^

A 2017 study published in the Annals of Surgery demonstrated the impact of VAs on the dissemination and visibility of scientific papers on Twitter. According to this study, each tweet containing the paper’s title had an average of 3,073 impressions (number of times people saw the tweet) and 11 retweets. When the same papers were published with VAs, each tweet averaged 23,611 impressions (7.7-fold increase; p <0.001) and 92 retweets (8.4-fold increase; p <0.001). In addition, tweets with only the paper’s title resulted in an average of 65.6 visits to the original paper, while VA tweets averaged 175.4 visits to the paper.^4^

Another 2017 study, which evaluated papers published in Molecules journal between March 2014 and March 2015; however, found different results. We evaluated 1,326 manuscripts published in the 13 volumes in the analyzed period, 760 without VA and 566 with a VA. Interestingly, papers published without VA performed better in terms of total downloads, summary views, and number of citations.^5^

Therefore, the effectiveness of VAs is not clear and there are already divergences in the still scarce literature on the subject. Notwithstanding the potential impact of this tool as an adjunct resource in the dissemination of scientific knowledge, VAs may have an intangible value as they represent a creative and innovative form of communication and interaction, reducing the formality of academia, and bringing younger readers closer to the journal. Clearly, further studies are needed to define the real effectiveness and the role of Vas; and certainly, some adjustments in form and content will be made in this process. Ancient folk wisdom says, “a picture is worth a thousand words.” If this proverb applies also in the academic context, we can expect new times in medical journals.
